# Carotid angiodysplasia complicated by the use of anti-hypertensive drugs during pregnancy: a case report

**DOI:** 10.1186/1752-1947-5-415

**Published:** 2011-08-25

**Authors:** Viviane Ribeiro de Paula, Laura Penna Rocha, Giovanni Carlos Tiveron, Camila Souza de Oliveira Guimarães, Marlene Antônia dos Reis, Beatriz Barco Tavares, Rosana Rosa Miranda Corrêa

**Affiliations:** 1 Discipline of General Pathology, Department of Biological Sciences, Universidade Federal do Triângulo Mineiro, Uberaba, Minas Gerais, Brazil; 2 Faculdade de Medicina de São José do Rio Preto, São José do Rio Preto, São Paulo, Brazil

## Abstract

**Introduction:**

Hypertensive syndromes in pregnancy are one of the leading causes of obstetric admissions into intensive care units. They are related to changes in the central nervous system caused by a decrease in cerebral perfusion pressure, indicated by an increase in intracranial pressure. These changes in pressure usually result from acute injuries or a decrease in the mean arterial pressure due to iatrogenic action or shock. However, other vascular disorders may contribute to similar occurrences.

**Case presentation:**

A 15-year-old girl was admitted to our hospital complaining of severe headaches since the eighth month of pregnancy, and presented with an arterial blood pressure of 180/120 mmHg. The diagnostic hypothesis was pre-eclampsia. Our patient's blood pressure levels remained elevated, and she was submitted to a cesarean section. After the procedure, she was referred to our infirmary, presenting with a blank distant look and with no interaction with the environment, dyslalia, and labial and upper and lower right limb paresis. She was confused and unable to speak, but responded to painful stimuli as she conveyed abdominal pain at superficial and deep palpation. The hypothesis of post-partum psychosis was suggested. She was then transferred to our intensive care unit, maintaining an impassive attitude in bed but reacting to external stimuli. Results of a computed tomography scan revealed ischemic infarction of the territory of her left middle cerebral artery. A selective cerebral arteriography showed bilateral occlusion of her internal carotid artery in the intracranial position, prebifurcation and angiodysplasia in the cervical segments of her internal carotid artery. Sixteen days after hospital admission, our patient died.

**Conclusion:**

This data shows the need for careful monitoring of hypertensive syndromes in pregnancy cases, especially in cases with a history of chronic hypertension or with vascular alterations, It also highlights the need for constant supervision of blood pressure levels during the use of anti-hypertensive medications.

## Introduction

Cardiovascular diseases during pregnancy are represented primarily by gestational hypertension syndromes and are an important cause of maternal morbidity, accounting for about 20-50% of obstetric admissions in the intensive care unit, and 15% of gestational deaths in Brazil [1,2].

Besides general systemic alterations, hypertensive syndromes in pregnancy (HSP) are related to changes in the central nervous system caused by the decrease of the cerebral perfusion pressure, indicated by the increase of the intracranial pressure. This can result from acute injuries or a decrease in the mean arterial pressure (MAP) due to iatrogenic action or shock. However, other vascular disorders may contribute to similar occurrences. The nervous system maintains cerebral autoregulation as a response mechanism to alterations in blood pressure levels. This adjusts the balance between cerebral perfusion pressure and cerebrovascular resistance, contributing to the perfusion of the nervous system, and also responsible for the balance of blood flow to vital organs [3].

In the current study we present the case of a pregnant woman with altered blood pressure levels, signs of loss of cerebral autoregulation and impairment of cerebral perfusion pressure, related to the manipulation of MAP and previous presence of carotid angiodysplasia.

## Case presentation

A 15-year-old girl was admitted to our hospital complaining of severe headaches since the eighth month of pregnancy, which had worsened in the last five hours. The following information was observed in the prenatal card: G1P0A0; gestational age: 37 weeks (estimated by date of last menstrual period); type O+ blood and negative serology. On examination our patient presented an arterial blood pressure of 180/120 mmHg, her uterine cervix was 20% effaced, with impervious external orifice and cephalic presentation. The diagnostic hypothesis was pre-eclampsia and routine investigations were requested to diagnose the HSP. After the tests, the diagnostic hypothesis changed to gestational hypertension. As our patient continued with elevated blood pressure levels, she was submitted to a cesarean section on the fourth day in hospital, delivering a male live baby with no intercurrences. After the procedure, she was referred to our infirmary, presenting a blank distant look and with no interaction with the environment. On examination she presented with dyslalia, and labial and upper and lower right limb paresis. During an examination six days after hospital admission, our patient was confused and unable to speak, but responded to painful stimuli as she conveyed abdominal pain at superficial and deep palpation. Moreover, she had a hematoma on the upper region of the surgical wound and physiological lochia. The hypothesis of post-partum psychosis was suggested and a careful neurological evaluation requested. She was administered the following drugs in hospital: nifedipine 1 mg, methyldopa 500 mg, 750 mg paracetamol, betamethasone, oxytocin, tenoxicam, promethazine, diclofenac sodium, cephalexin, and haloperidol. She was then transferred to the teaching hospital on the same day, and admitted to our intensive care unit, maintaining an impassive attitude in bed but reacting to external stimuli. She also emitted incomprehensible sounds, presented a blank look, with upward conjugated deviation of the eyes and mydriatic pupils reactive to light. She had Glasgow Coma Scale (GCS) score of nine points (2 +5 +2). Our patient was then submitted to orotracheal intubation with mechanical ventilation and central venous access through her right internal jugular vein, in addition to continuous sedation. Computed tomography (CT) of her skull and pelvis were requested. Results revealed tomography findings consistent with ischemic infarction of the territory of her left middle cerebral artery, but no pelvic alterations were observed. Our patient showed progressive worsening of the neurological symptoms, hyperthermia, tonic-extensor crisis, difficulty in breathing and scored four points in the GCS. A repeat skull CT revealed ischemic lesions in the mean cerebral system affecting her basal ganglia and parietal lobe, with proper filling of venous sinuses and no signs of meningeal inflammation. During this period, her blood pressure levels remained elevated (MAP 140-150 mmHg) and refractory to medication. In addition, our patient presented with neuropsychomotor agitation and periods of tachycardia and systemic arterial hypertension alternating with bradycardia and normotension. An urgent selective cerebral arteriography was requested; this showed bilateral occlusion of her internal carotid artery in the intracranial position, pre-bifurcation and angiodysplasia in the cervical segments of the internal carotid artery. Sixteen days after hospital admission, there was a worsening of our patient's condition and she died. Brain death as a consequence of bilateral obstruction of the internal carotid was certified as the cause of death.

## Discussion

In this case, our patient presented with elevated blood pressure levels and had an initial diagnostic of HSP. Although hypertension is one of the parameters for the diagnosis of HSP, other aspects must be considered; for example, the development of brain alterations, frequent in severe cases of HSP [4]. However, in this case, even with the administration of anti-hypertensive drugs and initial control of blood pressure levels, the brain alterations persisted.

Brain alterations are related to changes in MAP, but when they exceed the levels of regulation they are associated with a reduction of cerebral blood flow [3], being directly proportional to the cerebral perfusion pressure. Brain autoregulation is related to the ability to increase or decrease the metabolic demand, or to maintain the flow despite the increase or the reduction of the systemic blood pressure [5,6]. When the MAP exceeds the limits of autoregulation, the brain extracts more oxygen to compensate for the reduction in blood flow, so manifestations such as cerebral edema, reduction in the alertness level, migraine, mental confusion and anxiety, are observed in cases of failure in the mechanism of autoregulation [3,5,6]. High blood pressure for long periods of time, caused by chronic vascular lesions before pregnancy, influences the cerebral autoregulation mechanism, which, in this case, may have been affected by the use of anti-hypertensives. Some studies demonstrate that there must be a reduction of 25% in the MAP within the first hour [5]. However, reports on the duration of hypertension before pregnancy, the nature of the underlying disease before the treatment and monitoring of MAP reduction time were not found in our patient's medical records. This information could confirm that the action of the anti-hypertensive drugs was responsible for the cerebral alterations in our case.

Another diagnostic hypothesis related to the neurological alterations presented was post-partum psychosis. About 0.1-0.2% of psychiatric disorders during the post-partum period are represented by psychosis, characterized by the sudden onset of periods of psychotic mood with serious mental disorders within two to three weeks of birth. The classical symptoms are mental confusion, psychomotor agitation, anxiety and insomnia, evolving to manic, melancholy or even catatonic forms [7]. Despite the presence of similar symptoms, and the severity of our patient's clinical manifestations, especially prolonged low levels of consciousness characterized by low electrocardiogram scores, this hypothesis was rejected.

Carotid angiodysplasia was diagnosed by angiography as the underlying disease. Vascular malformation or angiodysplasia is characterized by endothelial hyperplasia and can be classified as hemangioma or as vascular malformation itself. Histologically, hemangiomas are characterized by endothelial hyperplasia during the proliferative phase, and by fibrosis, fatty infiltration and cell decrease in the involution phase. They are not present at birth, developing during the first months of life, and in 95% of cases they undergo a regression process that can be short or extend until the age of ten. As for malformations, the lesions are present at birth, grow at the same rate as the body and do not regress spontaneously. They consist of abnormal collections of blood vessels with the endothelium preserved and can be subdivided into arterial, venous and lymphatic vessels or a combination of them all [8,9]. Although hemangiomas regress at the age of ten, our patient's young age and the absence of an autopsy examination did not allow a clear distinction between the two forms of angiodysplasia in this case.

## Conclusion

The present study demonstrated a case of a hypertensive pregnant patient with carotid angiodysplasia and decompensation of cerebral autoregulation possibly caused by the use of anti-hypertensive drugs. This data shows the need for careful monitoring of HSP cases, especially in cases with a history of chronic hypertension or with vascular alterations, as well as constant supervision of the blood pressure levels during the use of anti-hypertensive medications.

## Consent Statement

Written informed consent was obtained from the patient's Father for publication of this case report and any accompanying images. A copy of the written consent is available for review by the Editor-in-Chief of this journal.

## Competing interests

The authors declare that they have no competing interests.

## Authors' contributions

VRP made significant contributions to the conception and acquisition of the data and was engaged in the design of the present study. LPR and CSOG participated in the sequence alignment and drafted the manuscript. BBT participated in the design and coordination of the study. MAR and RRMC were involved in drafting the manuscript and revising it critically for important intellectual content, providing general supervision for the research group and approved the final version to be published. All the authors read and approved the final manuscript.
